# Correction: LRRK2 dynamics analysis identifies allosteric control of the crosstalk between its catalytic domains

**DOI:** 10.1371/journal.pbio.3003017

**Published:** 2025-02-03

**Authors:** Jui-Hung Weng, Phillip C. Aoto, Robin Lorenz, Jian Wu, Sven H. Schmidt, Jascha T. Manschwetus, Pallavi Kaila-Sharma, Steve Silletti, Constanza Torres-Paris, Sebastian Mathea, Deep Chatterjee, Stefan Knapp, Friedrich W. Herberg, Susan S. Taylor

Constanza Torres-Paris is not included in the author byline. Constanza Torres-Paris should be listed as the ninth author and affiliated with the Department of Chemistry and Biochemistry, University of California, San Diego, California, United States of America. The contributions of this author are as follows: Data Curation, Formal Analysis, and Methodology.
