# Supplementary material for: LRRK2 dynamics analysis identifies allosteric control of the crosstalk between its catalytic domains
Source: PLoS Biol. 2022 Feb 22;20(2):e3001427. doi: 10.1371/journal.pbio.3001427 (PMC8863276; doi:10.1371/journal.pbio.3001427)
Supplement: S1 Fig — LRRK2, leucine-rich repeat kinase 2; ROC, ras-of-complex. (PDF) [file pbio.3001427.s001.pdf]

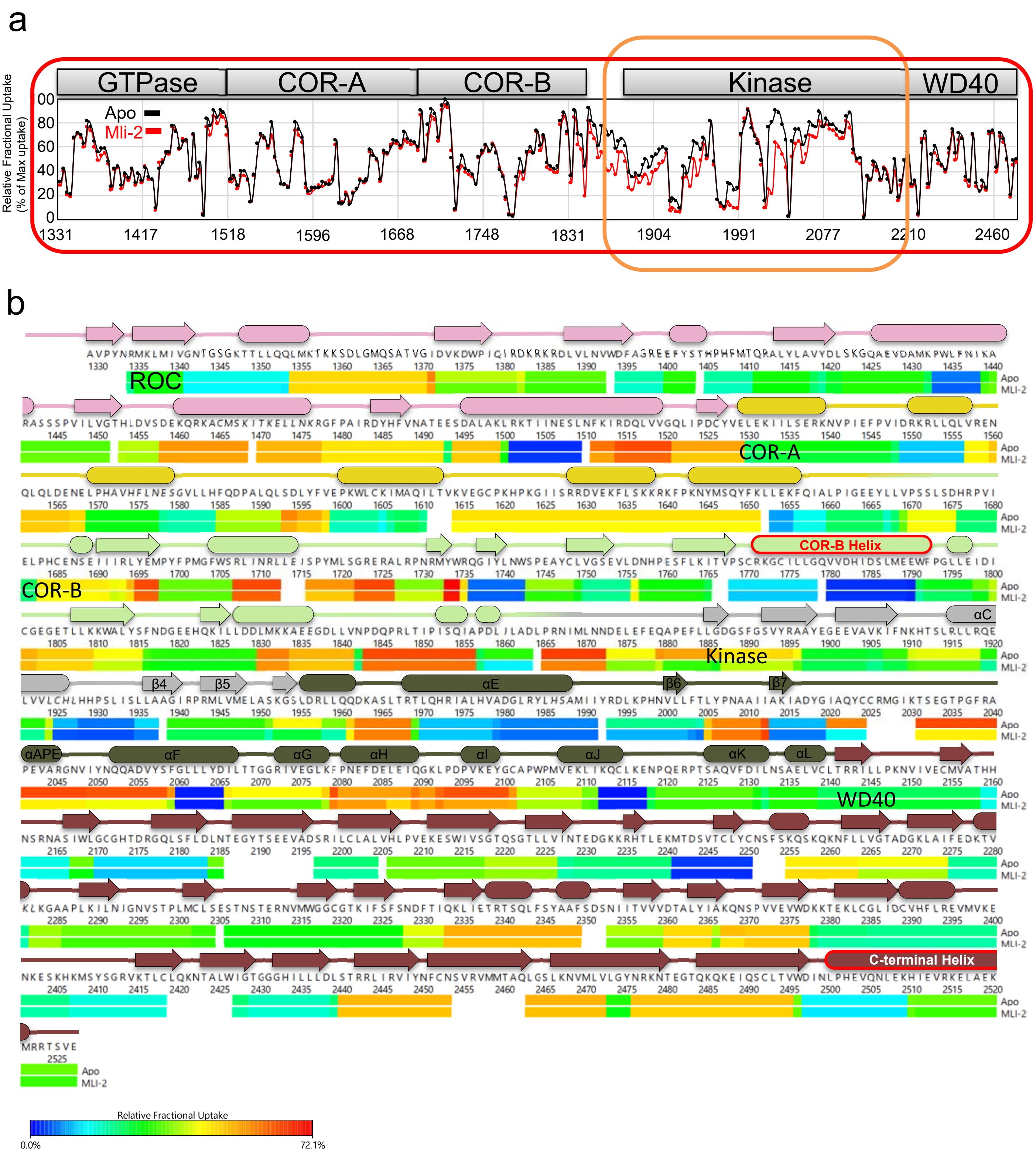

Figure S1. The deuterium uptake of LRRK2 in apo and MLi-2 bound conditions. (a) The relative deuterium exchange for each peptide detected from the N-terminal to C-terminal. The orange box highlights the kinase domain. (b) The heat map shows the relative fractional uptake by color at 2 min. Most of the high deuterium uptake regions are loops. Binding of MLi-2 reduces the deuterium uptake between COR-B, kinase and the activation loop.
