# Supplementary material for: LRRK2 dynamics analysis identifies allosteric control of the crosstalk between its catalytic domains
Source: PLoS Biol. 2022 Feb 22;20(2):e3001427. doi: 10.1371/journal.pbio.3001427 (PMC8863276; doi:10.1371/journal.pbio.3001427)
Supplement: S2 Fig — (PDF) [file pbio.3001427.s002.pdf]

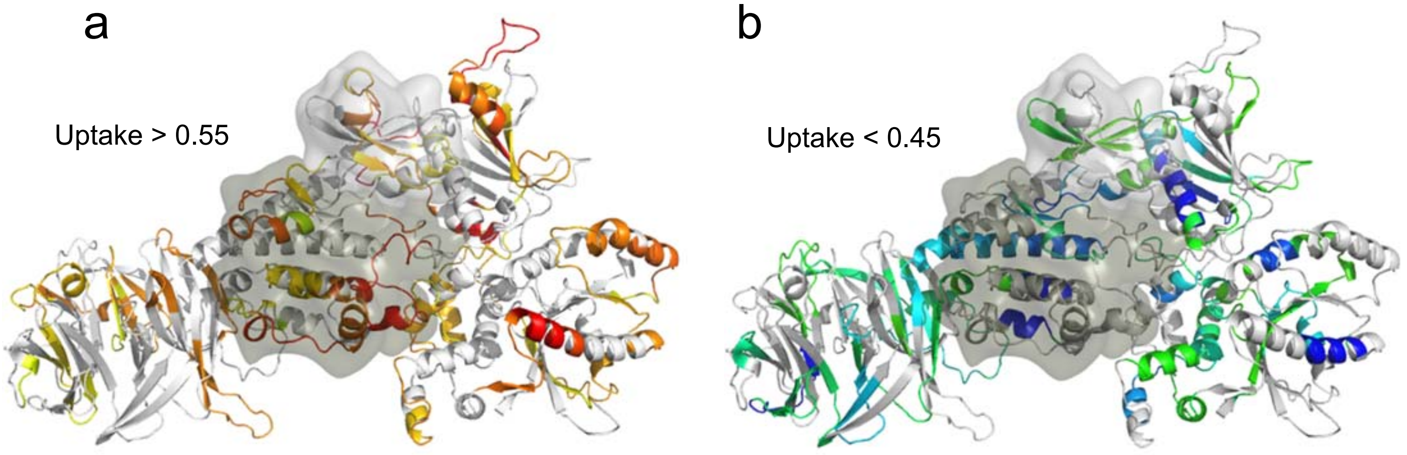

Figure S2. The deuterium uptake of LRRK2$_{RCKW.}$. (a) Regions where the relative fractional uptake was larger than 55%. This highlights the solvent exposed region located mainly at the surface or highly flexible regions such as the Activation Segment of the kinase domain. (b) Regions that are highly protected from the solvent are indicated by their relative fractional uptake deuterium uptake less than 45%.
