# Supplementary material for: LRRK2 dynamics analysis identifies allosteric control of the crosstalk between its catalytic domains
Source: PLoS Biol. 2022 Feb 22;20(2):e3001427. doi: 10.1371/journal.pbio.3001427 (PMC8863276; doi:10.1371/journal.pbio.3001427)
Supplement: S3 Fig — PMF, potential of mean force. (PDF) [file pbio.3001427.s003.pdf]

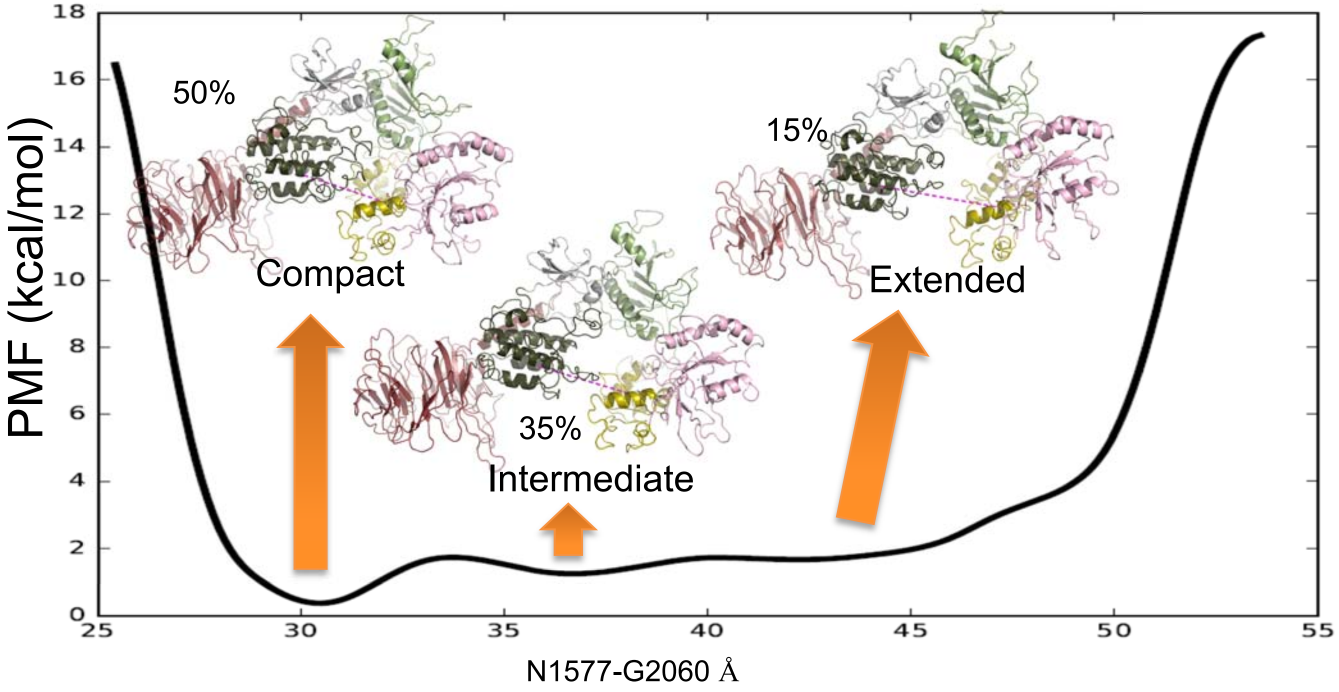


**Figure S3. LRRK2$_{RCKW}$ is fluctuated between compact and extended conformation.** The population of LRRK2$_{RCKW}$ in different conformations: compact (~50%); Intermediate (~35%) and Extended (~15%), measured by the distance between N1577 at the COR-A domain and the G2060 at the C-lobe of the kinase. PMF, potential of mean force.
