# Supplementary material for: LRRK2 dynamics analysis identifies allosteric control of the crosstalk between its catalytic domains
Source: PLoS Biol. 2022 Feb 22;20(2):e3001427. doi: 10.1371/journal.pbio.3001427 (PMC8863276; doi:10.1371/journal.pbio.3001427)
Supplement: S4 Fig — A-loop, activation loop; Ct-Helix, C-terminal helix; LRRK2, leucine-rich repeat kinase 2; ROC, ras-of-complex. (PDF) [file pbio.3001427.s004.pdf]

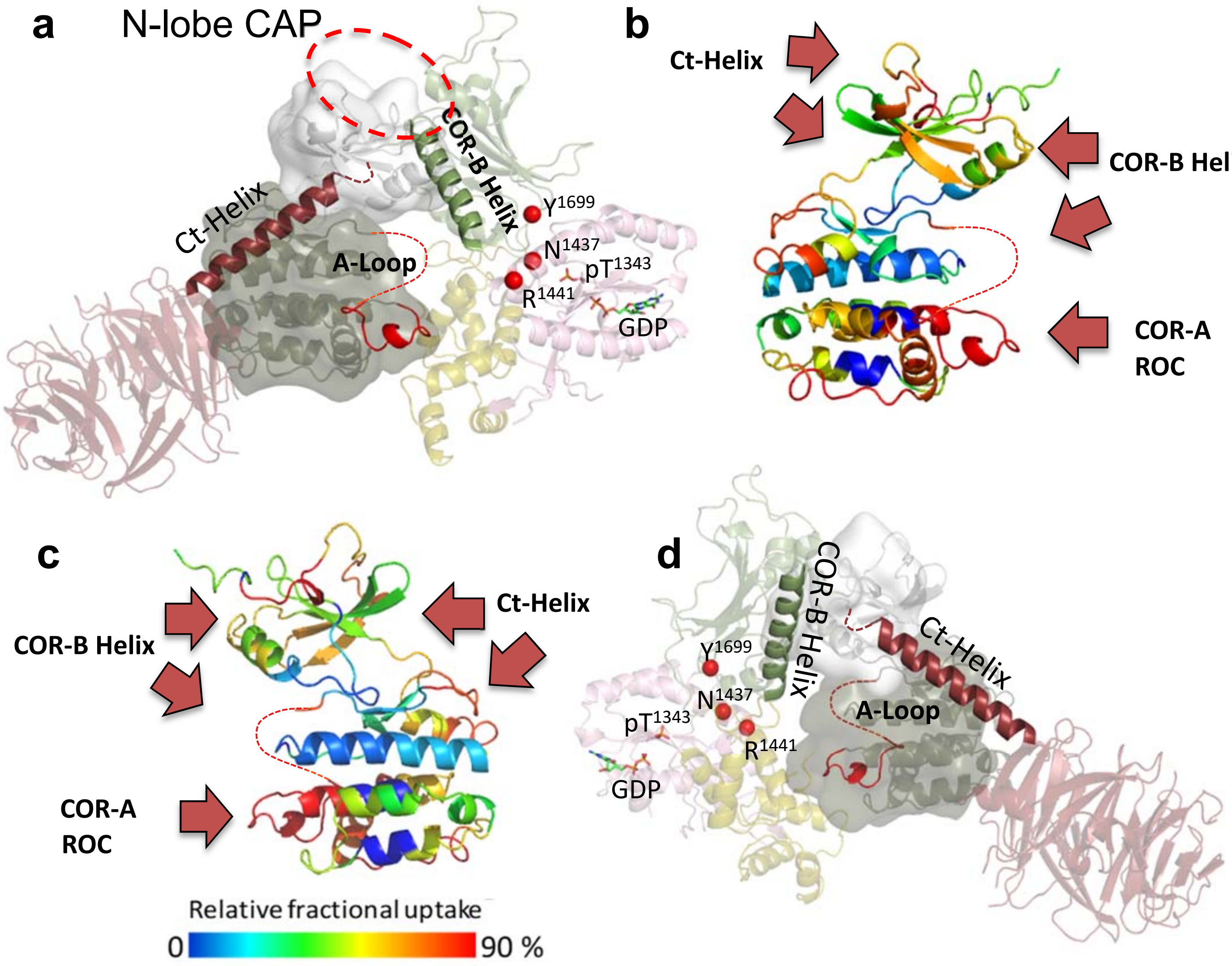


**Figure S4. The deuterium uptake of the kinase domain is influenced by the domains that flank the kinase domain of LRRK2.** (a) The deuterium uptake is plotted onto the kinase domain of LRRK2. The protection of the kinase domain and the dynamic properties of the kinase domain are affected by the COR-B Helix (teal) , Ct-Helix (red) and the COR-A domain. Organization of the kinase domain in $LRRK2_{RCKW}$ (b). Deuterium uptake of kinase domain (c) 180$^{o}$ rotation of panel (b). (d) 180$^{o}$ rotation of panel (a)
