# Supplementary material for: LRRK2 dynamics analysis identifies allosteric control of the crosstalk between its catalytic domains
Source: PLoS Biol. 2022 Feb 22;20(2):e3001427. doi: 10.1371/journal.pbio.3001427 (PMC8863276; doi:10.1371/journal.pbio.3001427)
Supplement: S5 Fig — A-loop, activation loop; GaMD, Gaussian accelerated molecular dynamics. (PDF) [file pbio.3001427.s005.pdf]

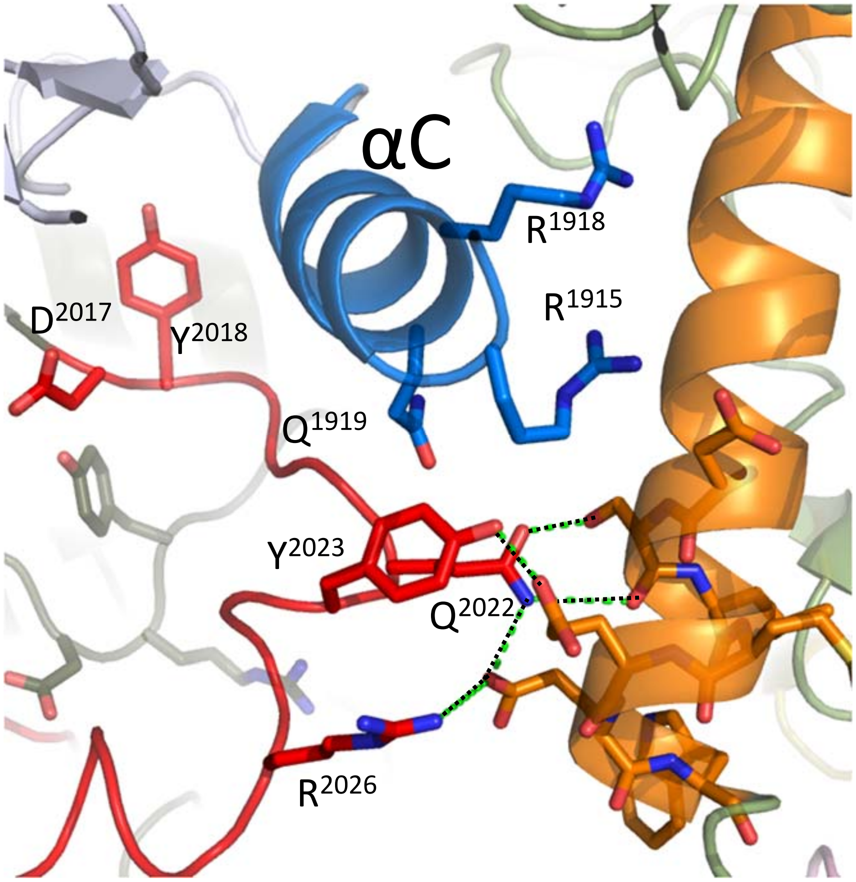

Figure S5. Interactions between Activation loop and COR-B Helix as shown with GaMD simulations. One snapshot of the GaMD simulations showing the residues on the A-Loop interacting with the COR-B Helix.
