# Supplementary material for: LRRK2 dynamics analysis identifies allosteric control of the crosstalk between its catalytic domains
Source: PLoS Biol. 2022 Feb 22;20(2):e3001427. doi: 10.1371/journal.pbio.3001427 (PMC8863276; doi:10.1371/journal.pbio.3001427)
Supplement: S6 Fig — Ct-Helix, C-terminal helix. (PDF) [file pbio.3001427.s006.pdf]

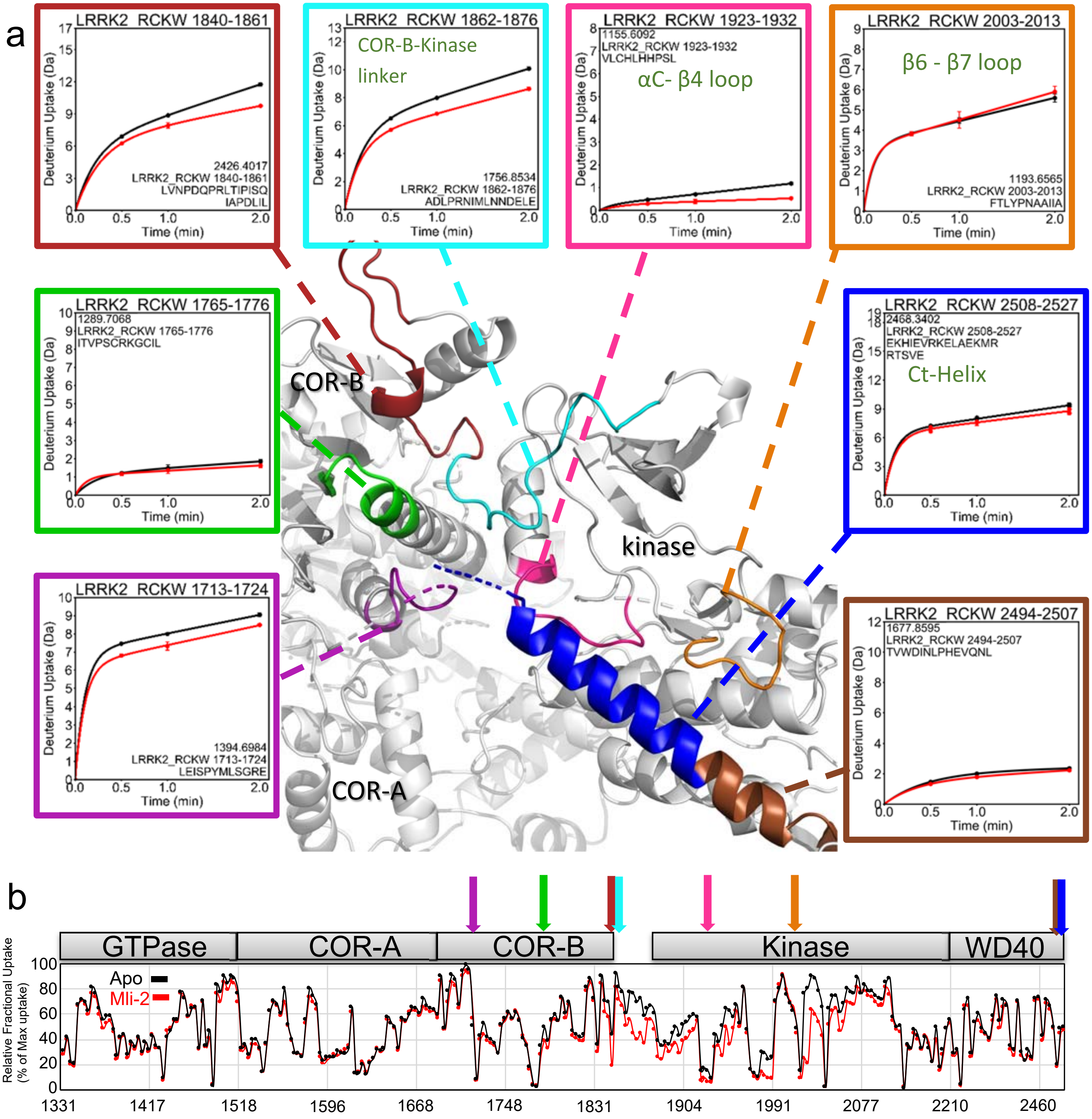


**Figure S6**. **Deuterium uptake around the Ct-Helix.** (a) The deuterium uptake of selected peptides is plotted and mapped on the $LRRK2_{RCKW}$ structure. The αC-β4 loop is shielded by the Ct-Helix, which resulted in low deuterium uptake. On the contrary, the β6-β7 loop of the kinase, the COR-B-kinase linker and the loop in COR-B domain show high deuterium uptake, indicating that they are solvent exposed. In the presence of MLi-2, these peptides demonstrated reduced deuterium uptake. (b) The relative deuterium exchange is visualized for each peptide with the arrows on top indicating the peptides shown in (a). The data underlying the graphs shown can be found in S1_Data.
