# Supplementary material for: LRRK2 dynamics analysis identifies allosteric control of the crosstalk between its catalytic domains
Source: PLoS Biol. 2022 Feb 22;20(2):e3001427. doi: 10.1371/journal.pbio.3001427 (PMC8863276; doi:10.1371/journal.pbio.3001427)
Supplement: S7 Fig — (PDF) [file pbio.3001427.s007.pdf]

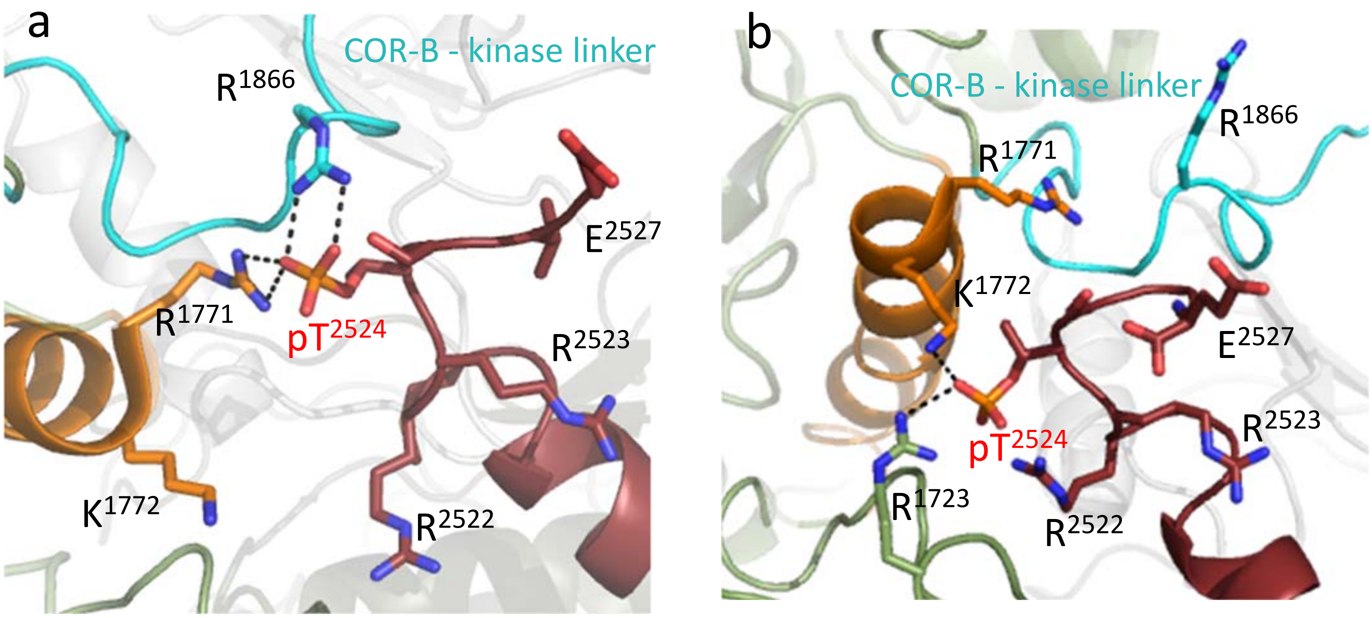

Figure S7. The interaction of pT2524. MD simulations show how the crosstalk between the C-terminus and COR-B domain can change when T2524 is phosphorylated or when 14-3-3 binds. Two snap shots of the simulations showing pT2524 interact with R1771 and R1886 (A) or K1772 and R1723 (B).
