# Supplementary material for: LRRK2 dynamics analysis identifies allosteric control of the crosstalk between its catalytic domains
Source: PLoS Biol. 2022 Feb 22;20(2):e3001427. doi: 10.1371/journal.pbio.3001427 (PMC8863276; doi:10.1371/journal.pbio.3001427)
Supplement: S8 Fig — AS, activation segment. (PDF) [file pbio.3001427.s008.pdf]

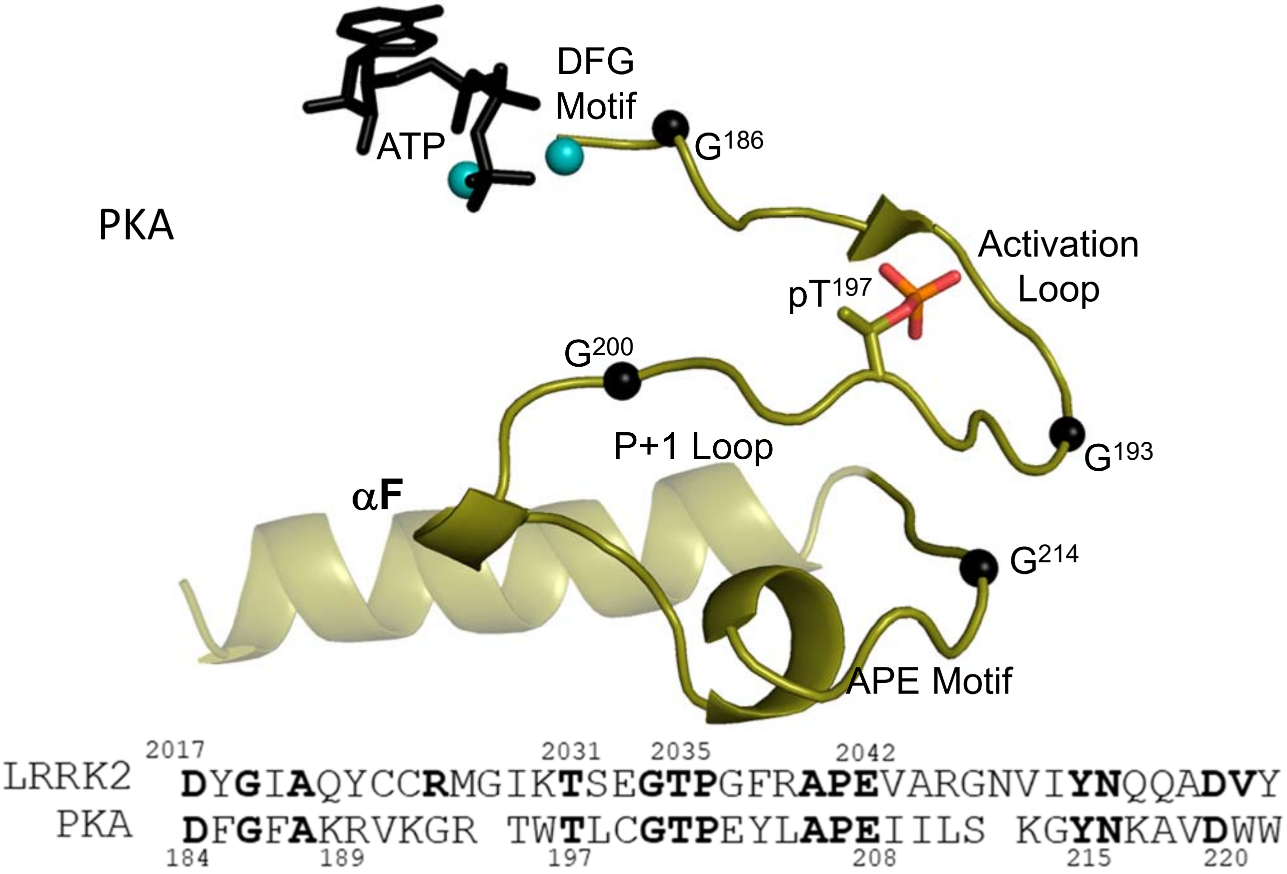

Figure S8. Activation Segments of PKA The motifs that are embedded in the AS of active PKA are summarized (PDB: 1ATP). The AS begins with the DFG motif and ends with the APE motif, two of the most highly conserved motifs in the protein kinase superfamily. In between these two motifs are the A-Loop and the P+1 Loop.
