# Supplementary material for: LRRK2 dynamics analysis identifies allosteric control of the crosstalk between its catalytic domains
Source: PLoS Biol. 2022 Feb 22;20(2):e3001427. doi: 10.1371/journal.pbio.3001427 (PMC8863276; doi:10.1371/journal.pbio.3001427)
Supplement: S9 Fig — A-loop, activation loop. (PDF) [file pbio.3001427.s009.pdf]

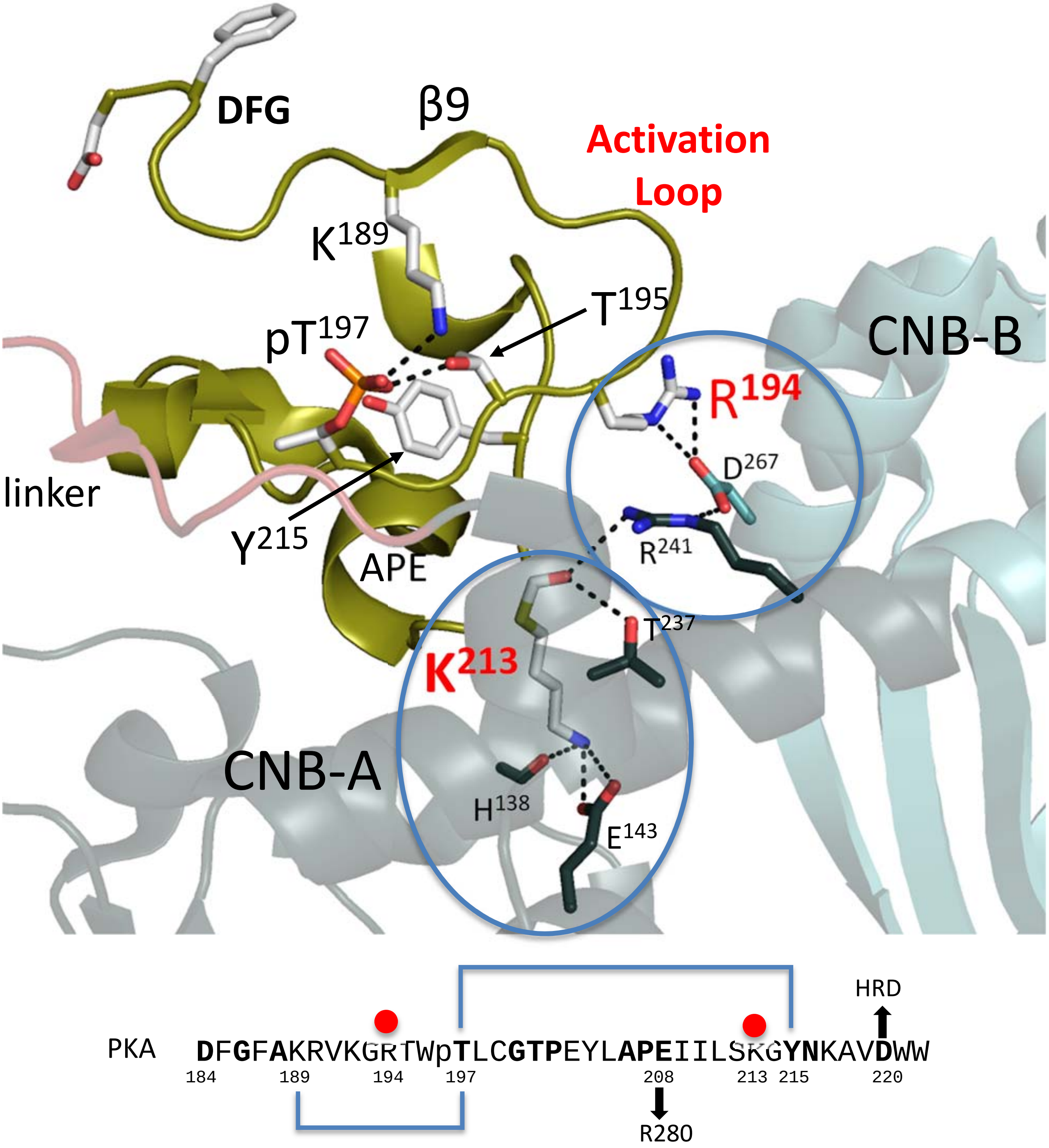

Figure S9. Interfaces created by the Activation Loop and the APE-αF Linker between the catalytic and regulatory subunits of PKA (PDB: 2QCS). The Activation Segment creates multiple surfaces that create docking sites for other proteins or domains. In the case of PKA the Activation Loop and the APE-aF linker interact with different parts of the RIa regulatory subunit.
