# Supplementary material for: LRRK2 dynamics analysis identifies allosteric control of the crosstalk between its catalytic domains
Source: PLoS Biol. 2022 Feb 22;20(2):e3001427. doi: 10.1371/journal.pbio.3001427 (PMC8863276; doi:10.1371/journal.pbio.3001427)
Supplement: S10 Fig — AS, activation segment; GaMD, Gaussian accelerated molecular dynamics; LRRK2, leucine-rich repeat kinase 2. (PDF) [file pbio.3001427.s010.pdf]

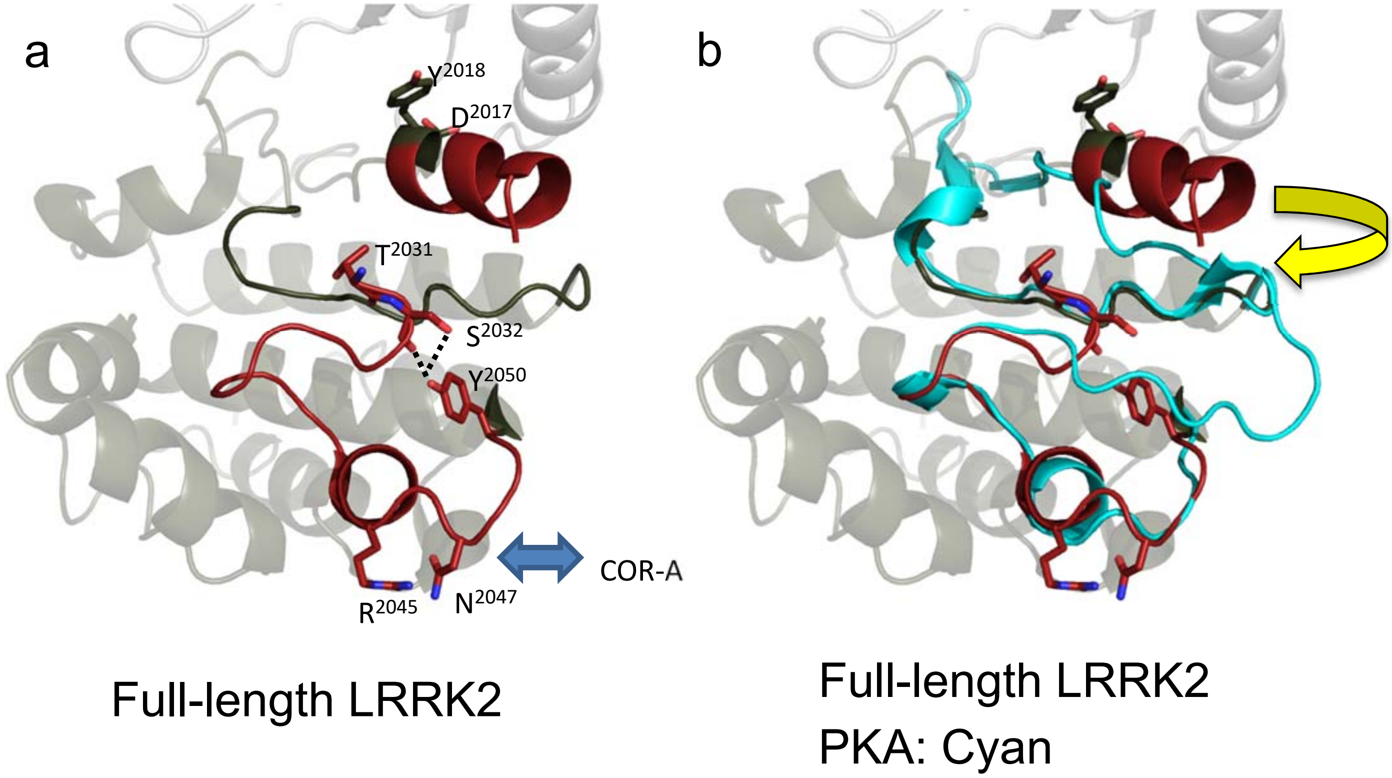

Figure S10. Activation Segment of inactive Full-length LRRK2 Aligned with active PKA. (a) The Activation Segment of LRRK2 (red) is mostly ordered in full-length LRRK2 (PDB: 7LI4). GaMD simulations suggest that R2045 and N2047 can interact with the COR-A domain. (b) Overlay with active PKA (cyan, PDB: 1ATP) shows good correlation after T2031 but the DFG motif in LRRK2 is in an inactive helical conformation that keeps the N-Lobe sequestered in an inactive conformation. This conformation resembles the inactive conformation of Src. When LRRK2 is in an active conformation the helix will transition to form a β-strand and the A-Loop will face towards the COR-B Helix as indicated with the yellow arrow.
