# Supplementary material for: LRRK2 dynamics analysis identifies allosteric control of the crosstalk between its catalytic domains
Source: PLoS Biol. 2022 Feb 22;20(2):e3001427. doi: 10.1371/journal.pbio.3001427 (PMC8863276; doi:10.1371/journal.pbio.3001427)
Supplement: S11 Fig — cryo-EM, cryogenic electron microscopy; LRRK2, leucine-rich repeat kinase 2; MD, molecular dynamics. (PDF) [file pbio.3001427.s011.pdf]

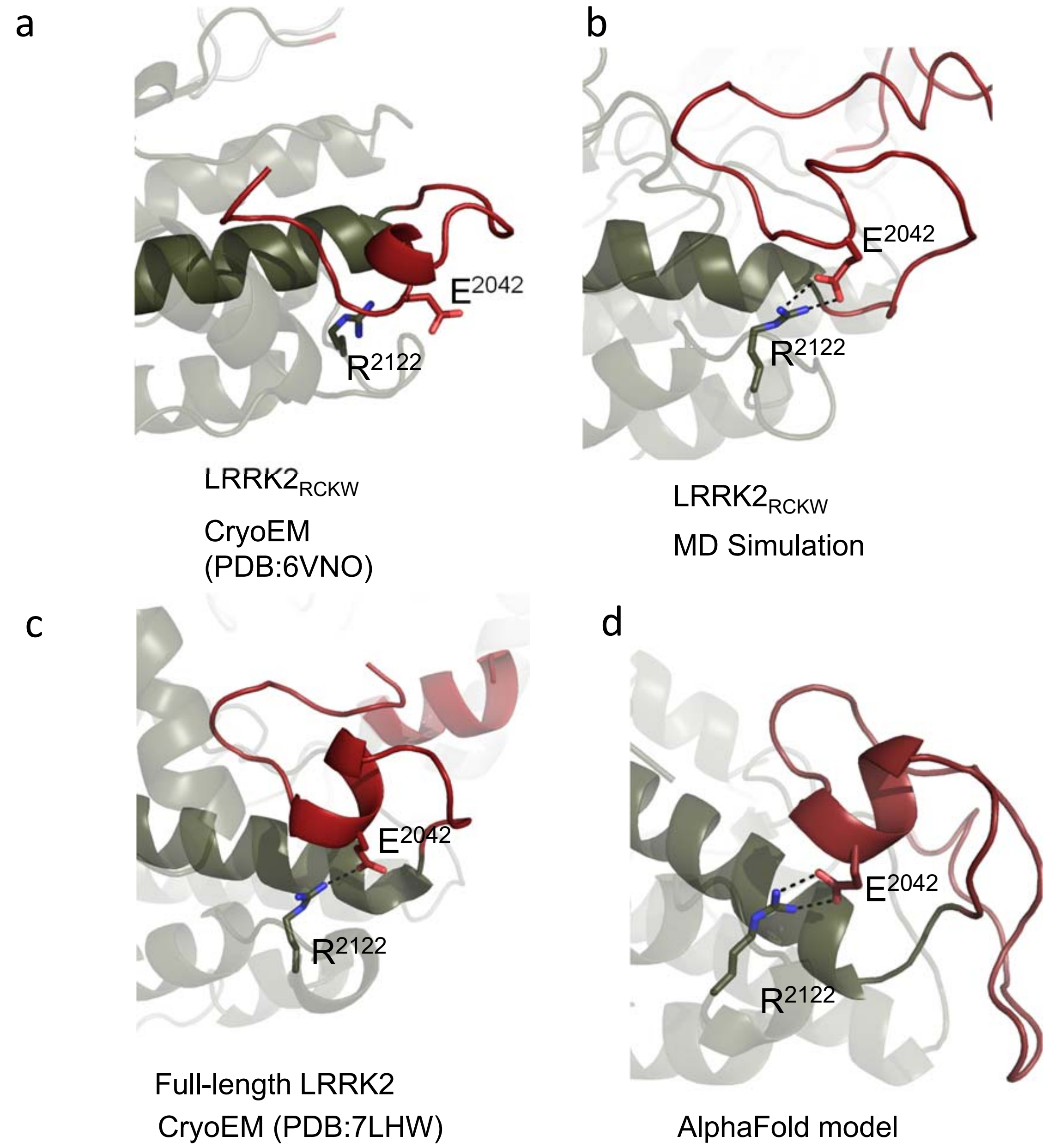

Figure S11. The APE motif is anchored to the αH-αI loop. (a) E2042 of the APE motif and R2122 on the αH-αI loop are close in $LRRK2_{RCKW}$ structure but not close enough for forming hydrogen bond. The interaction between E2042 and R2122 is identified in the MD simulation of $LRRK2_{RCKW}$ (b), in Full-length LRRK2 structure (c), and AlphaFold model (d).
