# Supplementary material for: LRRK2 dynamics analysis identifies allosteric control of the crosstalk between its catalytic domains
Source: PLoS Biol. 2022 Feb 22;20(2):e3001427. doi: 10.1371/journal.pbio.3001427 (PMC8863276; doi:10.1371/journal.pbio.3001427)
Supplement: S12 Fig — A-loop, activation loop; LRRK2, leucine-rich repeat kinase 2; MD, molecular dynamics; ROC, ras-of-complex. (PDF) [file pbio.3001427.s012.pdf]

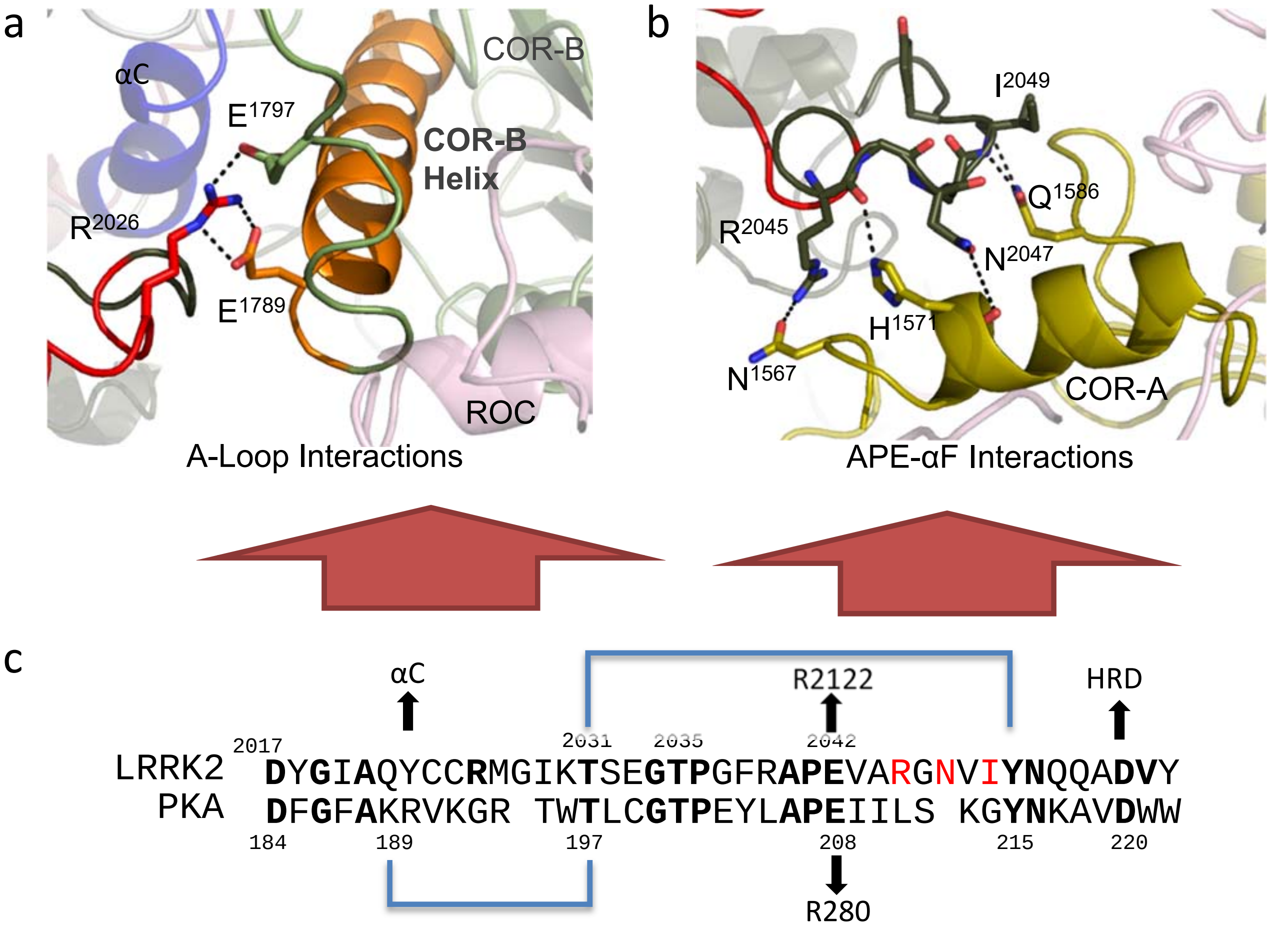

Figure S12. Activation loop is anchored by COR-A and COR-B domains. Interactions of the activation loop with COR-A domain and COR-B domain were identified in the MD simulation. (a) R2026 on the activation loop can interact with E1787 and E1797 on the COR-B domain. (b) The COR-A domain interacts with the APE-αF loop extensively to stabilize the activation loop. (c) The Activation Segment of LRRK2 and PKA.
