# Supplementary material for: LRRK2 dynamics analysis identifies allosteric control of the crosstalk between its catalytic domains
Source: PLoS Biol. 2022 Feb 22;20(2):e3001427. doi: 10.1371/journal.pbio.3001427 (PMC8863276; doi:10.1371/journal.pbio.3001427)
Supplement: S13 Fig — Ct-Helix, C-terminal helix; ROC, ras-of-complex. (PDF) [file pbio.3001427.s013.pdf]

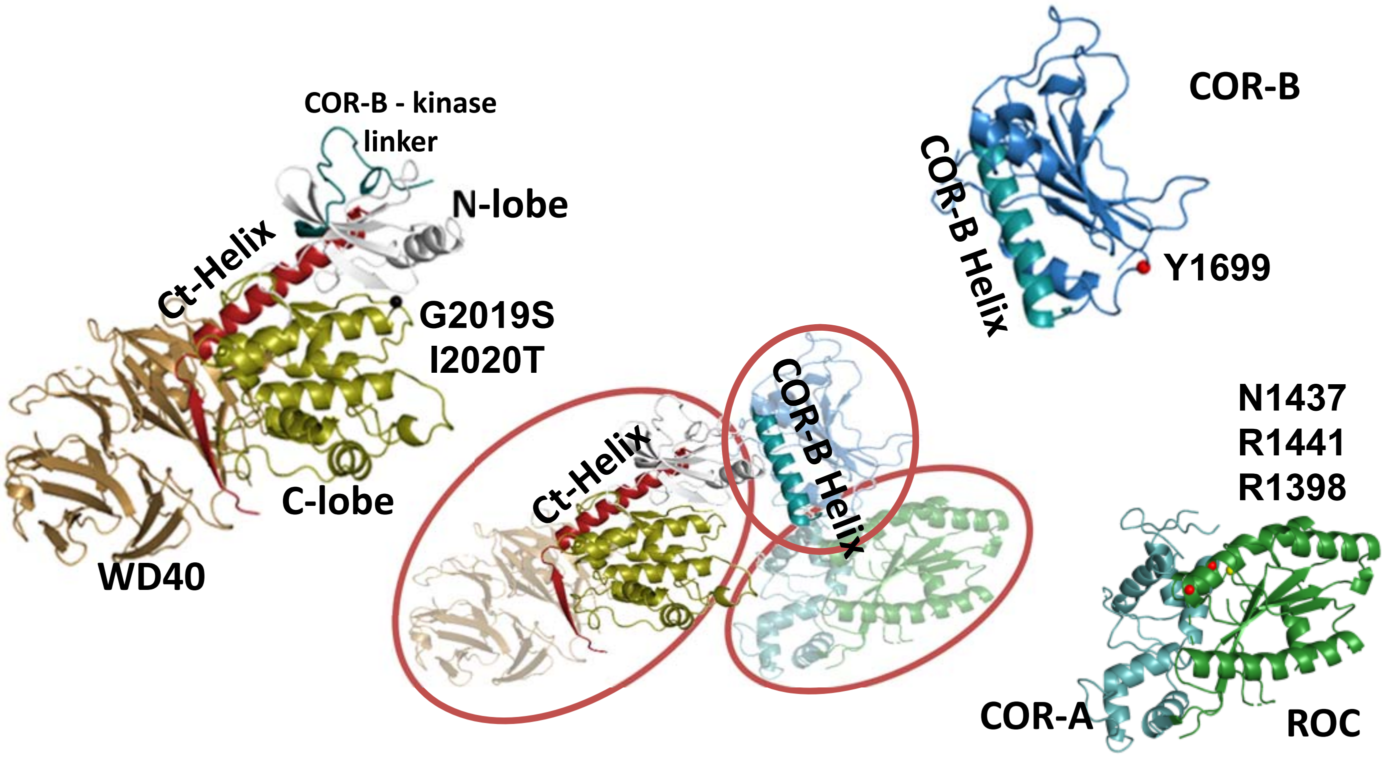

Figure S13. Three rigid bodies of LRRK2$_{RCKW.}$ Based on molecular dynamics., the domains of LRRK2$_{RCKW}$ can be grouped into three rigid bodies: 1. The ROC domain with the COR-A domain; 2. the COR-B domain with the kinase domain and the WD40 domain including the extended C-terminal helix and 3. the linker that joins COR-B to the kinase domain. The major pathogenic mutations are located at the interface between those rigid bodies and at the cleft that defines opening and closing of the kinase domain.
