# Supplementary material for: LRRK2 dynamics analysis identifies allosteric control of the crosstalk between its catalytic domains
Source: PLoS Biol. 2022 Feb 22;20(2):e3001427. doi: 10.1371/journal.pbio.3001427 (PMC8863276; doi:10.1371/journal.pbio.3001427)
Supplement: S14 Fig — A-loop, activation loop; Ct-Helix, C-terminal helix; ROC, ras-of-complex. (PDF) [file pbio.3001427.s014.pdf]

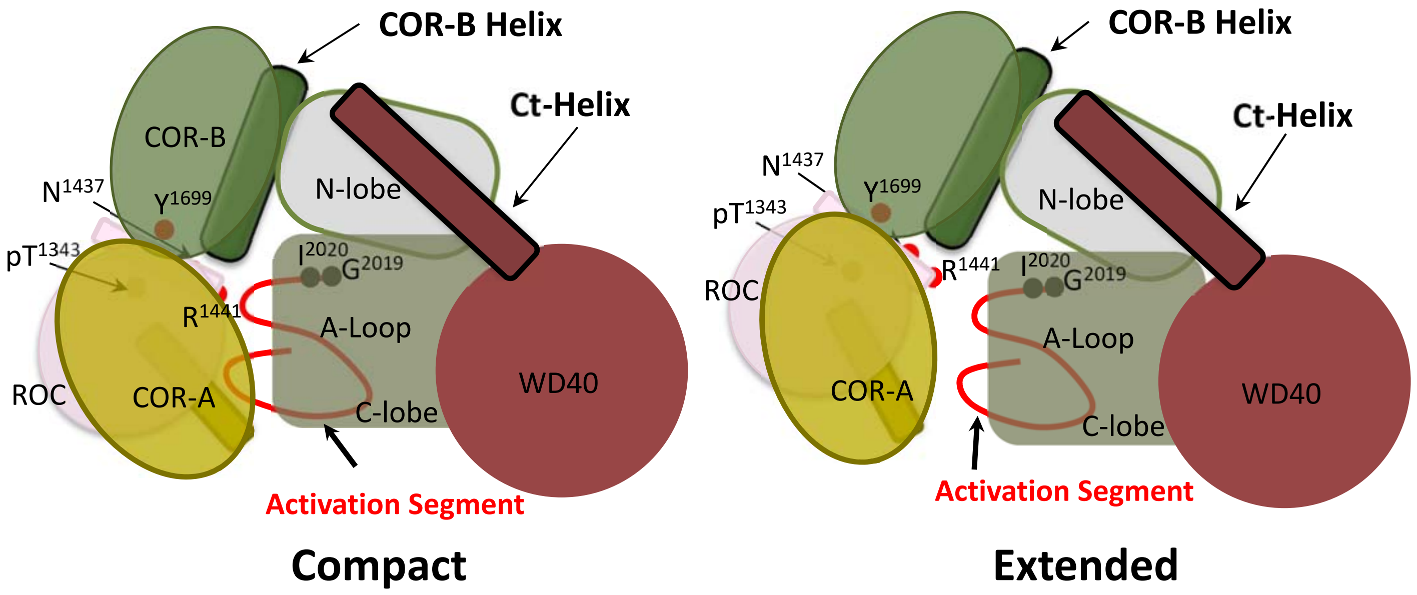

Figure S14. Domain contacts highlights the Ct-Helix interactions. Cartoon representation of the compact (left) and extended (right) states of $LRRK2_{RCKW.}$ The COR-B Helix and Ct-Helix are embracing the kinase domain while the COR-A domain only interacts with the C-lobe of the kinase in compact conformation. This view is rotated 180° from the view that is shown in Figure 8 and highlights the Ct-Helix.
