# Supplementary material for: LRRK2 dynamics analysis identifies allosteric control of the crosstalk between its catalytic domains
Source: PLoS Biol. 2022 Feb 22;20(2):e3001427. doi: 10.1371/journal.pbio.3001427 (PMC8863276; doi:10.1371/journal.pbio.3001427)
Supplement: Attachment — Submitted filename: PlosBiolReviewerResponse_1129.doc [file pbio.3001427.s021.doc]

9500 GILMAN DRIVE

SUSAN S. TAYLOR, Ph.D. LA JOLLA, CA 92093-0654

PROFESSOR, Chemistry and Biochemistry	(858) 534-3677 TELEPHONE

PHARMACOLOGY	(858) 534-8193 FACSIMILE

November 29, 2021

Dear Inesse,

We are very grateful for the reviews of our manuscript. The comments of the reviewers have been very constructive, and we have tried to address each of their specific concerns. As you indicate it would take a considerable amount of time to do the analysis of the R1441C mutant, and we agree with you that this would be beyond the scope of this manuscript. We have tried to respond to all the other comments, and we think that the revised manuscript is definitely improved. We are hoping it will now be suitable for publication. We are attaching the revised manuscript as well as our specific answers to the reviewers.

Thank you so much for handling our manuscript.

Sincerely,

Susan S. Taylor

Chancellor’s Distinguished Professor of Chemistry and Biochemistry

Distinguished Professor of Pharmacology
